# Postprandial Glucose and Gastrointestinal Hormone Responses of Healthy Subjects to Wheat Biscuits Enriched with L-Arginine or Branched-Chain Amino Acids of Plant Origin

**DOI:** 10.3390/nu14204381

**Published:** 2022-10-19

**Authors:** Amalia E. Yanni, Alexander Kokkinos, Panagiota Binou, Varvara Papaioannou, Maria Halabalaki, Panagiotis Konstantopoulos, Stamatia Simati, Vaios T. Karathanos

**Affiliations:** 1 Laboratory of Chemistry-Biochemistry-Physical Chemistry of Foods, Department of Nutrition and Dietetics, Harokopio University, 17671 Athens, Greece; 2 1st Department of Propaedeutic and Internal Medicine, Laiko General Hospital, School of Medicine, National and Kapodistrian University of Athens, 11527 Athens, Greece; 3 Division of Pharmacognosy and Natural Products Chemistry, Department of Pharmacy, National and Kapodistrian University of Athens, 15771 Athens, Greece; 4 Laboratory of Experimental Surgery and Surgery Research, School of Medicine, National and Kapodistrian University of Athens, 11527 Athens, Greece

**Keywords:** biscuit, legumes/seeds, L-arginine, branched chain amino acids, glucose responses, gastrointestinal hormones, appetite sensations

## Abstract

The study investigates the effects of wheat biscuits supplemented with plant flours originating from legumes/seeds enriched either in L-arginine (L-arg) or branched-chain amino acids (BCAAs) on postprandial glucose response of healthy subjects. Gastrointestinal hormone and amino acid responses as well as subjective appetite sensations are also evaluated. Subjects consumed wheat-based biscuits, enriched either in L-arg (ArgB) or BCAAs (BCAAsB) or a conventional wheat biscuit (CB) or a glucose solution (GS) in an acute randomized crossover design. Responses of glucose, insulin, ghrelin, glucagon-like peptide-1 (GLP-1), peptide YY (PYY) and glicentin, as well as those of L-arginine, L-leucine, L-isoleucine and L-valine, were evaluated over 180 min. Consumption of ArgB and BCAAsB elicited lower glucose iAUC compared to GS (*p* < 0.05). A lower iAUC for insulin was observed after consumption of BCAAsB (*p* < 0.05 compared to CB and ArgB), while ArgB elicited higher iAUC for GLP-1 accompanied by higher glicentin response (*p* < 0.05 compared to CB). BCAAsB and ArgB increased postprandial amino acid concentrations and caused stronger satiety effects compared to CB. Increasing protein content of wheat biscuits with supplementation of plant flours originating from legumes/seeds decreases postprandial glycemia and provides with healthier snack alternatives which can easily be incorporated into diet.

## 1. Introduction

Glucose responses after a meal are related with appetite and there is evidence from acute studies that low-glycemic foods or meals have higher satietogenic effect than high-glycemic foods or meals [1]. It is also known that high-glycemic index (GI) foods promote a more rapid return of hunger and increase subsequent energy intake [2]. However, the effects of fluctuations in glycemia and insulinemia on hunger and satiety remain debatable since there are studies which failed to support any effect [3]. The recently published results of the PREVIEW study, which compared the long-term effects of two diets, that of high protein (HP)-low GI vs. moderate protein (MP)-moderate GI on subjective appetite sensations during weight loss maintenance, showed that the HP-low GI diet suppresses hunger but not weight regain [4].

Proteins stimulate insulin secretion and reduce glucose concentration, depending on the amino acid profile of the ingested protein [5]. Amino acids, as products of protein digestion, hold pivotal roles in the mechanisms involved in peripheral and central regulation of energy intake. Enteroendocrine cells of the gut epithelial lining are directly activated by their exposure to the intraluminal content, driving the secretion of a number of gastrointestinal hormones as a sign of the nutrients’ arrival [6].

Both animal and plant proteins have been trending towards increased recognition in consumers’ minds; however, demand for healthier and sustainable nutrition drive research efforts to the development of plant protein products with enhanced protein quality. Plant proteins contain lower amounts of essential amino acids compared to animal proteins and are less digestible [7]. Strategies for combining plant proteins in order to meet indispensable amino acid requirements while also taking into consideration the critical roles of dispensable/conditionally indispensable amino acids can help to supply adequate high-quality protein, providing health benefits associated with increased intake of plant-based diets and reducing adverse environmental consequences. Legumes are a rich source of plant proteins and are suggested to moderate postprandial glucose levels [8,9]. Combining legumes, seeds and cereals can counter amino acid imbalance and enhance the presence of specific amino acids leading to the production of foods with functional properties which could positively affect postprandial glucose and appetite responses. 

Branched chain amino acids (BCAAs) and especially L-leucine (L-leu) activate key-signaling systems within hypothalamic neurons (mammalian targets of rapamycin/AMP-activated protein kinase, mTOR/AMPK) which regulate glucose metabolism and other essential physiological processes [10]. Studies in rodents have shown that L-leu suppresses food intake through regulation of neuropeptide Y/Agouti-related peptide (NPY/AgRP) and proopiomelanocortin (POMC) release [11,12,13,14]. On the other hand, L-arginine (L-arg), a conditionally indispensable amino acid, is the endogenous precursor of the potent vasodilator nitric oxide and promotes insulin-mediated glucose uptake [15]. Oral supplementation with L-arg in lean and obese mice increased GLP-1 and insulin concentrations and improved glucose clearance [14]. Additionally, studies in rodents and recently in humans revealed that L-arg increases postprandial circulating levels of GLP-1 and PYY and reduces food intake [16,17,18]. 

Biscuits enjoy high popularity in the human diet and can be excellent snack alternatives as well as potential carriers of ingredients with glucose- and appetite-regulating properties [19,20]. However, common wheat biscuits elicit high glucose responses and have poor protein quality [21]. In the present study, wheat biscuits supplemented with plant flours originating from legumes and seeds were developed and primarily examined regarding their effects on postprandial glucose concentration. Plant flours have been combined in a way to achieve the enrichment of wheat biscuits with amino acids which have been associated with glucose and appetite regulation, more precisely, either BCAAs or L-arg. In this context, beyond the effects on glucose which was the primary outcome, additional analysis was performed regarding the effects of biscuits’ consumption on gut hormone responses and appetite sensations.

## 2. Materials and Methods

### 2.1. Subjects

Procedures involving human subjects were carried out in the Diabetes Laboratory of the 1st Department of Propaedeutic and Internal Medicine, Laiko General Hospital, Athens School of Medicine, in collaboration with the Laboratory of Chemistry-Biochemistry-Physical Chemistry of Foods, Department of Nutrition and Dietetics, Harokopio University of Athens, Greece. The study was approved by the Institutional Review Board/Ethics Committee of Laiko General Hospital and the Bioethics Committee of Harokopio University. The study was registered in clinicaltrials.gov as NCT03974165.

Thirty healthy subjects (15 males, 15 females), mean age 26.5 ± 5.3 years with body mass index (BMI) 25.7 ± 4.6 kg m^−^^2^ were recruited by poster advertisements and personal communication. Subjects who were currently modifying diet or exercise patterns to lose weight or with known food allergies or intolerances, restrictive eaters, excessive exercisers or trained athletes, as well as candidates taking any medications or food supplements, were excluded. Pregnant, breast feeding and postmenopausal women were also excluded. 

Before entering the study, participants were informed about the time and efforts required, gave voluntary informed written consent for participation and completed questionnaires related to diet, exercise and lifestyle habits. 

### 2.2. Test Foods

Two different wheat-based biscuits were designed, with the same protein content originating from plant flours (legumes and seeds) but with different amino acid compositions especially regarding BCAAs (BCAAsB) and L-arg (ArgB). More specifically, BCAAsB had higher BCAAs content while ArgB higher L-arg content. A common wheat biscuit (CB) with the same basic recipe was also prepared. Flours originating from legumes which are used in the Mediterranean cuisine, such as white beans and lentils as well as seeds such as millet and sesame, were examined regarding their amino acid composition. Flours were combined in a way to achieve the enrichment of the final products with the specific amino acids as well as to differentiate BCAAs and L-arg content between the two types of biscuits. The other amino acids and dietary fiber content remained as close as possible. The composition of flours of the test biscuits (final product) to achieve desired amino acids content was 28.8% wheat flour, 18.9% millet flour, 4.7% white bean flour and 10.5% yellow lentil protein concentrate (which consists of 55% protein on dry basis) for BCAAsB. For ArgB the composition was 41.2% wheat flour, 11.8% defatted sesame flour and 5.9% yellow lentil flour. CB contained only wheat flour (57.4% in the final product). The biscuits were prepared by a food manufacturer (ELBISCO S.A., Greece) and flours were supplied by the manufacturer’s partners.

Nitrogen (protein: Nx6.25) was measured by Kjeldahl (ISO 1871) [22] and fat by Soxhlet procedures. Total, soluble and insoluble dietary fibers were determined by the AOAC method 991.43 [23]. The available carbohydrates of the samples were calculated by difference. Amino acids were separated by ion exchange chromatography and determined by reaction with ninhydrin using photometric detection (EU 152/2009) [24]. Energy content (kJ) was calculated according to the following equation: Energy = 17 × (g protein + g carbohydrate) + 37 × (g fat) + 8 × (g total dietary fibers). 

### 2.3. Study Design and Experimental Protocol

The study was designed as an acute, single-blind randomized controlled cross-over study with the three test biscuits (BCAAsB, ArgB, CB) and a solution containing 50 g of glucose as reference food (GS), provided in random order with an in-between washout period of 1 week. Randomization was performed using a computer-generated randomization schedule. Subjects arrived at the Diabetes Laboratory of the 1st Department of Propaedeutic and Internal Medicine of Laiko University Hospital between 08:00 and 09:00 after having fasted overnight. On arrival, subjects underwent anthropometric and baseline blood pressure measurements. Body weight was measured in the fasted state with light clothing on a scale (Tanita WB-110MA, Tokyo, Japan) and height using a stadiometer (Seca Mode 220, Germany). Body fat was determined by bioelectrical impedance analysis (Tanita WB-110MA, Tokyo, Japan). Waist circumference was measured at the midpoint between the lower margin of the least palpable rib and the top of the iliac crest in a standing position at the end of gentle expiration. Hip circumference measurement was taken around the widest portion of the buttocks. Blood pressure was performed by conventional methods. After 10 min of rest, subjects were asked to fill out the first set of visual analogue scales (VAS) questions assessing hunger, fullness and desire to eat. Then, an intravenous catheter was placed in a forearm vein and baseline samples were drawn. Subjects were then given one of the four test meals to consume within 15 min. Biscuit amounts yielding 50 g of available carbohydrates were served with 250 mL of tap water. Glucose solution contained 50 g of glucose (anhydrous) diluted in 250 mL water. Blood samples were collected and VAS were filled at 15, 30, 45, 60, 90, 120 and 180 min after food ingestion. 

Participants were instructed to eat a meal of their preference the previous evening and repeat it prior to each study session. Twenty-four-hour dietary recalls and a physical activity questionnaire were collected for the day before each study visit. Participants did not exercise regularly and were highly encouraged to maintain their regular lifestyle habits throughout the entire experimental period.

### 2.4. Blood Analyses

#### 2.4.1. Biochemical Parameters and Gastrointestinal Hormones 

Blood samples were collected in K_3_EDTA-coated vacutainers and centrifuged immediately (1000× *g* for 10 min at 4 °C) for plasma separation. For serum, blood was collected in plain tubes, allowed to clot at room temperature for 30 min and then centrifuged (1000× *g* for 10 min at 4 °C). After isolation, plasma and serum were stored at −80 °C until further analysis.

Glucose concentrations at 0, 15, 30, 45, 60, 90, 120 and 180 min were determined in plasma by an electrochemical method in an automated analyzer (ΥSI 2300 STAT PLUS, YSI Incorporated, Yellow Springs, OH, USA). Basal biochemical measurements were performed in serum, at the beginning of the study, on an automated biochemical analyzer (Medilyzer, Medicon Hellas S.A., Athens, Greece) using commercially available diagnostic kits.

For the determination of ghrelin, plasma was pre-treated as previously described [25]. Ghrelin at 0, 30, 60, 120 and 180 min was assayed by a sandwich ELISA method on a microtiter plate reader (Bio-Rad Model 680 Microplate Reader, Bio-Rad Laboratories, Athens, Greece) using a commercially available human ghrelin kit (Human Ghrelin Total ELISA kit, Merck-Millipore, Burlington, MA, USA). Total GLP-1, PYY and glicentin were also measured in plasma at 0, 30, 60, 90, 120 and 180 min by sandwich ELISA methods using commercially available kits (Human Total GLP-1 kit, Merck-Millipore, Burlington, MA, USA; Human PYY (Total) ELISA kit, Merck-Millipore, Burlington, MA, USA and Glicentin ELISA, AnshLabs, Webster, TX, USA, respectively). Insulin was measured in serum at 0, 30, 45, 60, 90, 120 and 180 min (Human Insulin ELISA kit, Merck-Millipore, Burlington, MA, USA). 

#### 2.4.2. Plasma Amino Acids

##### Sample Preparation

A total of 40 μL of plasma was placed inside a 0.5 mL tube and 10 μL of 10% sulfosalicylic acid was added for protein precipitation. After vortexing and centrifugation for 2 min at 10,000× *g*, 10 μL of the supernatant was transferred into a new tube and mixed with 40 μL of labelling buffer. The samples were vortexed and centrifuged again and 10 μL of the supernatant were mixed by vortex with 5 μL of aTRAQ^®^ labelling reagent. Samples were incubated at room temperature for 30 min and at the end of the incubation, and 5 μL of hydroxylamine were added. Samples were vortexed and then dried for 45 min with no temperature applied. The dried samples were reconstituted to 32 μL of the 2.5 μL/L aTRAQ^®^ internal standard. 

##### Separation and Detection

Liquid chromatography analysis on an Acquity UPLC^®^ H Class system from Waters company (Milford, MA, USA) system (Waters) Detection was performed on an AB Sciex TripleTOF^®^ 5600^+^ mass spectrometer from AB Sciex company (Framingham, MA, USA) equipped with a DuoSpray™ ion source operated in the positive ESI mode. Separation was achieved on a C18 column (Amino Acid Analyzer C18 Reversed Phase 5 µm, 4.6 mm × 150 mm, Sciex), using a gradient containing water (mobile phase A) and methanol (mobile phase B), both containing 0.1% formic and 0.01% heptafluorobutyric acid. Column temperature was maintained at 50 °C and the flow rate was set to 0.8 mL/min. The run time, injection-to-injection, was 22 min. 

The analytes’ determination was carried out with a Swath acquisition method with 12 variable *m*/*z* windows (accumulation time 0.075 s) and a full scan mass experiment (accumulation time 0.25 s). Declustering potential was set to 30, while collision energy (CE) was set to 18 V for up to 150 *m*/*z* analytes and to 30 V for the higher masses, with a spread of 5 V. For the ESI source, temperature was set to 600 °C and ion spray voltage was 4500 V. Source gas and exhaust gas were set to 60 psi while curtain gas was set to 30 psi. Data acquisition was performed using the Analyst^®^ 1.7.1 software (AB Sciex, Framingham, MA, USA) and spectral interpretation was performed using the Sciex OS software platform (AB Sciex, Framingham, MA, USA). The variable windows for the Swath method resulted from the Sciex’s Swath Variable Window Calculator.

##### Identification and Quantitation

Quantification was achieved by dividing the analyte area by the IS area and then multiplying by the IS concentration. The full scan spectrum was used for analytes’ quantification while for the identification both the full scan spectrum and the spectrum MS/MS were used, as well the elution time of each amino acid in comparison with its respective internal standard. Additionally, a library with pure MS/MS spectra was created with the implementation of Information Dependent Acquisition (IDA) methods to standard solutions for aTraq amino acid derivatives and their corresponding Internal Standards in order to enhance the identification confidence of the analytes with %Fit Scores of samples’ spectrums in comparison to the standard solutions’ ones.

##### Method Validation

The method was validated in accordance with the EMEA/CHMP/EWP/192217/2009 Rev. 1 Corr. 2. [26]. The protocol’s performance was assessed for the parameters of linearity, carry over, selectivity, limit of quantitation and upper limit of quantitation, precision and accuracy. The precision and accuracy parameters were evaluated through a complete analysis of Certified Reference Material, a plasma sample with known amino acid concentrations analyzed and certified by the aTRAQ Kit for Amino Acid Analysis of Physiological Fluids constructor (AB SCIEX).

### 2.5. Subjective Satiety Measures

Study participants were provided with VAS booklets in order to assess appetite and satiety regarding the four different meals. VAS were filled before meal and then at 15, 30, 45, 60, 90, 120, and 180 min postprandially just before blood samples were obtained, in order to reduce the stress of sampling. Three main questions were included; “How hungry do you feel”, “How full do you feel” and “How great is your desire to eat.” A 10 cm line scale ranging from 0 (“not at all”) to 10 (“extremely”) expressing the most negative and the most positive rating, respectively, was used. Subjects were instructed to place a vertical mark on the line to correspond with their feelings and were not allowed to discuss their ratings with each other or refer to the previous ratings when marking the scale. Quantification was made by measuring the distance in cm from the left end of the line to the mark. 

### 2.6. Sensory Evaluation

Sensory evaluation of the three biscuits was performed in order to investigate the effect of different composition on organoleptic characteristics. Twenty-five trained testers participated. Specifically, appearance, color intensity, texture, odor, taste, aftertaste and overall acceptance were evaluated on a 9-point hedonic scale (1 = extremely dislike, 5 = neither like or dislike, 9 = like extremely). The sensory evaluation took place at the manufacturer’s facilities.

### 2.7. Calculation and Statistical Analysis

AUCs of glycemic and insulinemic responses were calculated applying the trapezoidal rule using the incremental AUC (iAUC), ignoring area under the baseline [27]. AUC for ghrelin was expressed as decrease from preprandial values and was calculated using iAUC ignoring area above the *x* axis, while AUCs for GLP-1, PYY, glicentin and amino acids (L-arg, L-leu, L-ile and L-val) were expressed as increase from preprandial values and were calculated using iAUC ignoring area below the *x* axis. Accordingly, iAUCs for VAS were estimated. 

Descriptive statistics are presented as mean ± SD and results as mean ± SEM. ANOVA for repeated measures, followed by Bonferroni’s post hoc test was used to compare the postprandial changes in blood variables and subjective appetite ratings between treatments and identify significant differences at specific time points as well as differences between iAUC values. Regarding sensory characteristics, one-way ANOVA was applied.

*p* < 0.05 was considered statistically significant. The SPSS 21.0 (IBM SPSS Statistics for Windows, Version 21.0, Armonk, NY, USA: IBM Corp) statistical software package was used for the analysis.

## 3. Results

### 3.1. Subjects 

All subjects completed the four sessions of the study and no adverse reactions were reported. Subjects’ characteristics are presented on Table 1. Classic biochemical parameters were within normal range. 

Participants complied with the requirements of the study including continuation of dietary and physical exercise habits and maintenance of consistent body weight throughout the entire experimental period.

### 3.2. Test Food Analysis

Nutritional composition of each test biscuit and amino acids content are presented in Table 2. The total amino acids content (TAA) was similar in BCAAsB and ArgB. However, BCAAsB was about 11.5% richer in BCAAs and about 8.3% in indispensable amino acids (IAA) compared to ArgB, which was approximately 28.9% richer in L-arg. Aromatic amino acid (AAA) concentrations were similar in the two biscuits. Both BCAAsB and ArgB were at least 50% richer in TAA, IAA and AAA compared to CB. 

### 3.3. Blood Analyses

#### 3.3.1. Glucose and Hormone Responses

No significant treatment × weight interactions were detected after the application of a linear mixed model, with patients fitted at random; iAUC of each parameter was considered as a dependent variable and treatment and weight as fixed factors.

**Figure 1:**
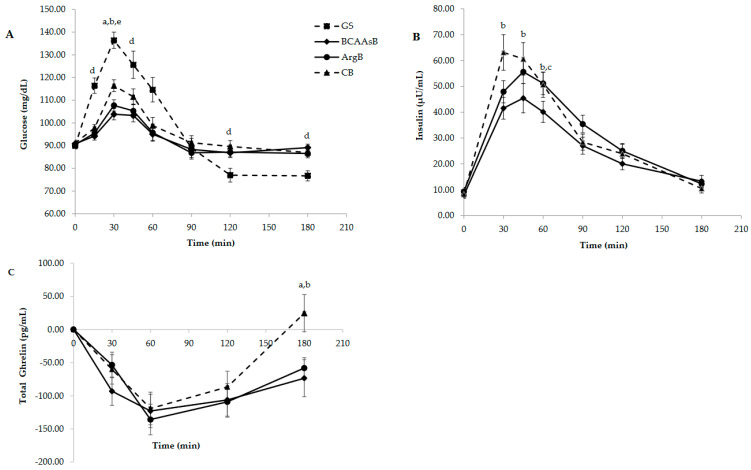

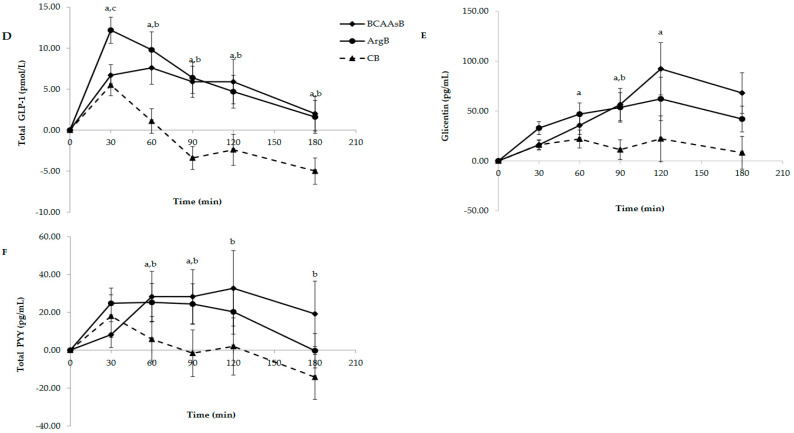
Responses of glucose (**A**), insulin (**B**), ghrelin (**C**), GLP-1 (**D**), glicentin (**E**) and PYY (**F**) of subjects, 3 h after the consumption of the three tested biscuits. Values are expressed as mean ± SEM (*n* = 30). ^a^ *p* < 0.05 between ArgB and CB, ^b^ *p* < 0.05 between BCAAsB and CB, ^c^ *p* < 0.05 between BCAAsB and ArgB, ^d^ *p* < 0.05 between GS and ArgB, BCAAsB, CB, ^e^ *p* < 0.05 between GS and BCAAsB, ArgB.

The highest mean peak plasma glucose concentration was caused by GS followed by CB, ArgB and BCAAsB and was observed 30 min after ingestion of the tested foods (Figure 1A). Glucose concentrations after BCAAsB and ArgB were lower compared to GS and to CB (*p* < 0.05). A significant difference between ArgB, BCAAsB and CB compared to GS was noticed at all the rest time points i.e., 15, 45, 60, 120 and 180 min postprandially (*p* < 0.05). GS also exerted the highest iAUC value followed by CB, ArgB and BCAAsB while BCAAsB and ArgB caused lower glucose iAUC compared to CB and to GS (*p* < 0.05, Table 3). All the iAUCs referred to time duration 0–180 min. 

BCAAsB elicited a milder insulinemic response compared to the other two biscuits (Figure 1B). Specifically, insulin concentration was lower at 30 and 45 min compared to CB (*p* < 0.05) and at 60 min compared to CB and ArgB (*p* < 0.05). iAUC caused by BCAAsB was also significantly lower compared to those caused by CB and ArgB (*p* < 0.05 for both biscuits).

Total ghrelin concentrations remained at low levels after the ingestion of the three biscuits reaching significant difference after BCAAsB and ArgB compared to CB at 180 min (Figure 1C, *p* < 0.05). No differences between iAUCs were observed.

GLP-1 levels were increased after ingestion of BCAAsB and ArgB at all examined time points, compared to CB (*p* < 0.05, Figure 1D). iAUC was significantly higher after ingestion of ArgB compared to CB (*p* = 0.001) while the difference between BCAAsB and CB iAUCs was marginally significant (*p* = 0.054, Table 3). Glicentin response is presented in Figure 1E. There was an increase after ArgB consumption at 60, 90 and 120 min and after BCAAsB at 90 min compared to CB (*p* < 0.05). Glicentin iAUC was higher after ArgB compared to CB (*p* = 0.009) while the difference between BCAAsB and CB was marginally significant (*p* = 0.093, Table 3).

PYY remained at elevated levels after BCAAsB and ArgB consumption with significantly increased concentrations after BCAAsB at 60, 90, 120 and 180 min and after ArgB at 60 and 90 min compared to CB (*p* < 0.05, Figure 1F). No differences between the PYY iAUCs were noticed.

#### 3.3.2. Amino Acid Responses

**Figure 2:**
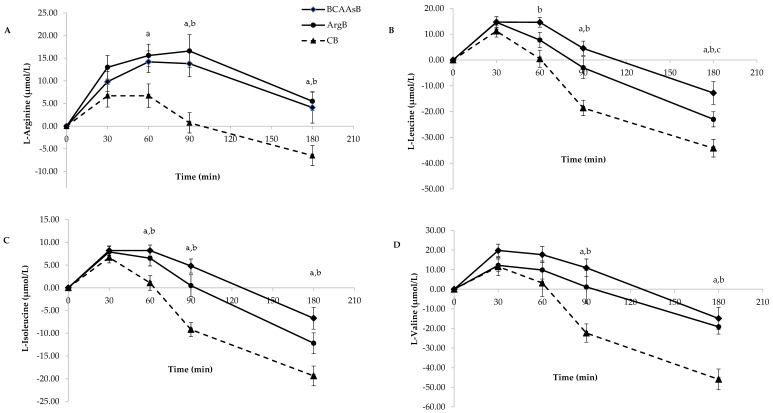
Responses of L-arginine (**A**), L-leucine (**B),** L-isoleucine (**C**) and L-valine (**D**) of subjects 3 h after the consumption of the three tested biscuits. Values are expressed as mean ± SEM (*n* = 30). ^a^ *p* < 0.05 between ArgB and CB, ^b^ *p* < 0.05 between BCAAsB and CB, ^c^ *p* < 0.05 between BCAAsB and ArgB.

The postprandial responses of the four amino acids of interest, specifically L-arg, L-leu, L-ile and L-val after the ingestion of the three biscuits are presented in Figure 2. 

ArgB ingestion caused a significant increase in plasma concentration of L-arg at 60, 90 and 180 min and BCAAsB ingestion at 90 and 180 min postprandially compared to CB (*p* < 0.05, Figure 2A). Both iAUCs caused by ArgB and BCAAsB were higher (*p* < 0.05, Table 4). 

L-leu concentrations were significantly elevated compared to CB at 60, 90 and 180 min after ingestion of BCAAsB and at 90 and 180 min after ingestion of ArgB (*p* < 0.05, Figure 2B). iAUC caused by BCAAsB was significantly higher compared to that of CB (*p* < 0.05). 

Both biscuits caused higher L-ile concentrations at 60, 90 and 180 min postprandially as well as significantly higher iAUCs compared to CB (*p* < 0.05, Table 4, Figure 2C).

BCAAsB and ArgB also increased L-val concentrations compared to CB (*p* < 0.05 at time points 90 and 180 min for both biscuits, Figure 2D) but no differences between iAUCs were observed (Table 4). 

**Figure 3:**
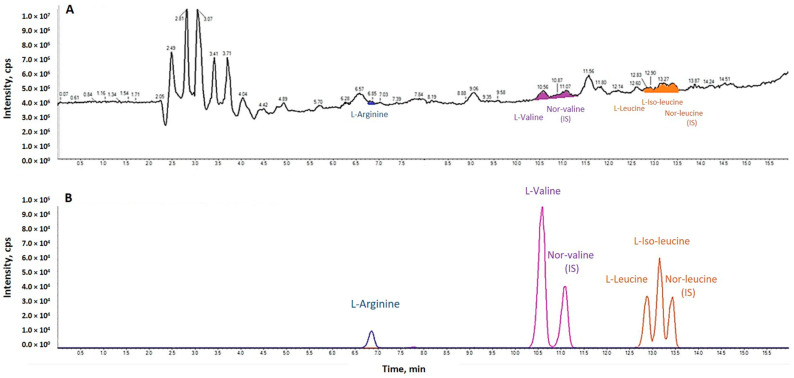
Total Ion Current Chromatograph of a sample of the study integrating the analyzed amino acids and two Internal Standards (**A**). Extracted Ion Chromatograph of L-arginine, L-valine, nor-valine (Internal Standard), L-leucine, L-isoleucine and nor-leucine (Internal Standard) (**B**).

A typical ion chromatograph of a plasma sample is presented in Figure 3.

### 3.4. Subjective Satiety Measures

**Figure 4:**
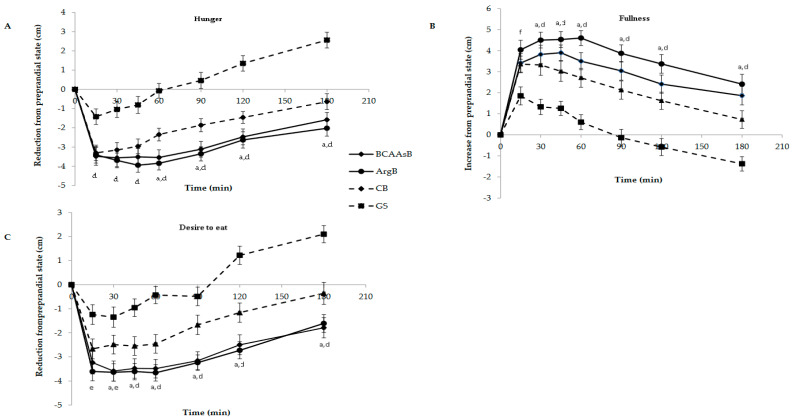
Mean subjective appetite rating differences from preprandial values of hunger (**A**), fullness (**B**) and desire to eat (**C**) of subjects after ingestion of the four test meals. Values are expressed as mean ± SEM (*n* = 30). ^a^ *p* < 0.05 between ArgB and CB, ^d^ *p* < 0.05 between GS and ArgB, BCAAsB, CB, ^e^ *p* < 0.05 between GS and BCAAsB, ArgB. ^f^ *p* < 0.05 between GS and ArgB.

Subjective appetite rating differences from preprandial values over the 180 min after the consumption of the three biscuits and GS are illustrated in Figure 4. Significantly lower hunger ratings were noticed after the consumption of the three biscuits at all examined time points compared to GS (*p* < 0.05). ArgB caused lower values at 60, 90, 120 and 180 min also compared to CB (*p* < 0.05). Hunger iAUCs of the three biscuits were significantly decreased compared to GS while that of ArgB was also lower compared to that of CB (*p* < 0.05, Figure 4A). Regarding fullness ratings, these were higher after BCAAsB, ArgB and CB consumption compared to GS (*p* < 0.05) at time points 30, 45, 60, 90, 120 and 180 min. ArgB caused significantly higher values at 30, 45, 60, 90, 120 and 180 min also compared to CB (*p* < 0.05, Figure 4B). Both enriched biscuits elicited higher fullness iAUCs compared to CB (*p* < 0.05, Table 3). As far as desire for the next meal was concerned, all three biscuits caused lower values at 45, 60, 90, 120 and 180 min compared to GS (*p* < 0.05). ArgB consumption showed significant effects also compared to CB at all time points after 45 min (*p* < 0.05, Figure 4C) while BCAAsB showed at 90 and 180 min. Both enriched biscuits caused significantly lower iAUCs compared to CB and all biscuits compared to GS (*p* < 0.05, Table 3). 

### 3.5. Sensory Evaluation

Different composition of the biscuits had a significant effect on some organoleptic characteristics. In particular, color intensity was different between the enriched samples and the control sample (*p* < 0.05) in contrast to their appearance, which did not seem to be affected (Table 5). Regarding textural characteristics, hardness of CB was higher and gumminess lower (*p* < 0.05) compared to BCAAsB and ArgB. Furthermore, no differentiation was observed in terms of odor of the evaluated samples, while the control biscuit appeared to be characterized by a clearly stronger taste, compared to the other two biscuits. Overall acceptance showed that both biscuits were more preferred than the control biscuit.

## 4. Discussion

The overwhelming prevalence of overweight and obesity, a central player in the pathophysiology of many non-communicable diseases such as type 2 diabetes, dyslipidemia and atherosclerosis, is considered a major threat to human health [28,29]. Understanding the role of nutrients in the mechanisms involved in glucose regulation and activation of gastrointestinal hormones may provide vital insights for controlling food intake and prove to be useful for effective obesity prevention. 

Proteins reduce blood glucose concentration through the stimulation of insulin secretion, depending on their amino acid profile [5]. Additionally, they increase the levels of specific anorectic peptides to a greater extent than other macronutrients thus mediating satiety and facilitating weight loss [30,31,32,33]. 

Legumes, beyond their protein content, are suggested to moderate glycemic response due to the higher amount of resistant starch and amylose and the delayed carbohydrate absorption [9,34,35,36]. Attempts to substitute wheat flour for legume flours have been proven successful in ameliorating postprandial glucose responses to foods with high carbohydrate content, such as bread and bread products [9,37].

In the present study two types of wheat-based biscuits supplemented with plant flours originating from legumes and seeds, one higher in BCAAs and one higher in L-arg, were developed and examined regarding their effects on postprandial glucose responses. In addition, insulin, gastrointestinal hormones and subjective appetite ratings were evaluated. In order to shed light on the underlying mechanisms, responses of the amino acids of interest (L-arg, L-leu, L-ile, L-val) were examined. Sensory evaluation was also performed. 

Enrichment with specific amino acids was obtained by using flours originating from common legumes and seeds of the Mediterranean cuisine (white beans, lentils, millet and sesame). The main effort was the enrichment of the final products through the addition of a combination of plant flours with high content of these amino acids. The target was to differentiate BCAAs and L-arg content between the two types of biscuits and keep as close as possible the content of total/other amino acids, macronutrients and dietary fibers, thus enabling the distinction of postprandial effects to be attributed to BCAAs or L-arg. 

Both biscuits, BCAAsB and ArgB, caused a lower glycemic response compared to the common wheat biscuit and to the reference food (glucose solution). BCAAsB also elicited a lower insulinemic response than that of ArgB and CB, whereas ArgB consumption led to higher insulin levels similar to those caused by CB. This could be attributed to the high L-arg content of ArgB (~29% higher than that of BCAAsB). L-Arg exhibits strong insulinotropic characteristics and these results are in accordance with previous studies which have shown that substitution of wheat flour for legume flour rich in L-arg resulted in lower GI but also elicited a higher insulin response [36].

Consumption of the three biscuits caused a reduction in total plasma ghrelin concentration, which remained at low levels until 120 min postprandially. BCAAsB and ArgB consumption contributed to maintenance of low hormone concentrations until 180 min, reaching statistical significance compared to CB at this time point. Both biscuits helped subjects to maintain lower concentrations of ghrelin for a longer time.

GLP-1 iAUC after ArgB consumption was significantly higher compared to CB while BCAAsB also caused a higher iAUC with marginally significant difference. Both biscuits enhanced plasma L-arg response. L-arg is a GLP-1 secretagogue [16] and possibly the higher intake of this amino acid induced a higher response of GLP-1. It has been reported that L-arg is sensed by amino acid sensors in the gut which affect gut hormone release [37] and that some of these receptors are expressed in the same cells which express gut hormones [38,39]. However, it should be taken into consideration that this effect could be attributed to a synergistic role of L-arg with other amino acids such as L-leu or other nutrients in general. 

In addition to GLP-1, glicentin is another proglucagon peptide which is also secreted by L-cells in response to food intake and recent studies have shown that similar to GLP-1, glicentin exerts insulinotropic effects [40]. Glicentin actions are not fully understood but there is a potential interest in this hormone as a factor implicated in metabolic diseases such as diabetes and obesity. The glicentin peptide contains the entire sequence of glucagon, glicentin-related pancreatic peptide (GRPP) and oxyntomodulin and assessing its concentration accurately without cross-reacting with the other peptides was difficult until recently. For this reason, literature on circulating glicentin is poor [40].

The results of the present study revealed that both ArgB and BCAAsB caused a postprandial increase in glicentin response after ingestion, inducing stronger effects which remained for a longer time than those of the common wheat biscuit. Significantly higher levels of the hormone were observed at certain time points. iAUC after ArgB consumption was significantly higher compared to CB while BCAAsB also caused a higher iAUC with marginally significant difference. The role of specific amino acids in glicentin response has not been evaluated so far except for one older study [41]. Effects of L-arg or BCAAs either alone or synergistically with other nutrients on postprandial glicentin responses are challenging to evaluate, especially considering the fact that these responses have been associated with prediction of weight loss [42,43]. 

Plasma concentrations of BCAAs remained at higher levels for a longer time after consumption of BCAAsB. BCAAs act in the brain as signals of dietary protein intake. L-Leu suppresses food intake and influences signaling systems associated with feeding behavior [13] and regulation of glucose metabolism [8]. Additionally, BCAAs and especially L-ile upregulate intestinal and muscular glucose transporters, enhancing glucose consumption and utilization [44]. However, elevated circulating levels of BCAAs have been observed in obese individuals and are associated with insulin resistance and type 2 diabetes mellitus. It seems that elevated BCAAs are rather a marker than a cause of insulin resistance and reflect abnormal BCAA metabolism, but this relationship is under investigation [45,46].

Consumption of both biscuits caused stronger feelings of satiety compared to the control biscuit as revealed after evaluation of their effects on subjective appetite sensations. Specifically, BCAAsB and ArgB elicited significantly higher iAUCs for “fullness” and lower iAUCs for “desire for the next meal” compared to CB. ArgB consumption exerted also a significantly lower iAUC for “hunger” compared to that of CB. Increasing the number of questions and performing VAS measurements in different sessions from those of blood sampling could provide additional, clearer information. The two biscuits had very good organoleptic characteristics, were well-tolerated by the subjects and were not less preferred than the control biscuit.

The evaluation of hormone responses led to better conclusions regarding the satiating effect of the enriched biscuits. However, a factor which has to be taken into consideration is that amino acids produce a slowing down of stomach emptying that could explain, in part, the observed metabolic responses. Certainly, intragastric administration of L-leu and L-ile in healthy men lowers postprandial blood glucose, at least in part by slowing gastric emptying [47]. Additionally, studies in Sprague-Dawley rats have shown that L-arg and other amino acids inhibit and delay gastric emptying as evaluated by a breath test using [1-^13^C] acetic acid [48].

Beyond amino acid and carbohydrate content, differences in energy intake could account for the differences in subjective appetite and gut hormone responses. Since the primary outcome of the study was to examine the effects on glucose concentrations, biscuit meals with the same content of available carbohydrates were provided to the subjects. In addition, the provided amount of carbohydrates affects GLP-1 and different carbohydrates’ loads would lead to different GLP-1 responses. Indeed, ingestion of carbohydrates causes a rapid increase in circulating GLP-1 levels within 30–60 min in healthy humans. Glucose and fructose increase GLP-1 secretion in a dose-dependent manner and a possible mechanism involves the closure of ATP-sensitive K_ATP_ channels and subsequent membrane depolarization in GLUTag cells. Membrane depolarization results in Ca^2+^ influx, which induces vesicular exocytosis and secretion of GLP-1 into the circulation [49,50].

It has to be reported that ArgB was further examined in a long-term study conducted in subjects with overweight/obesity following a hypocaloric diet. The results showed that participants who consumed the ArgB exerted significantly higher body weight reduction and lower energy intake compared to the participants who consumed isocaloric amounts of the control wheat biscuit [51]. Animal studies have shown that L-arg has a delayed and sustainable anorectic effect [18]. Beyond the above-mentioned results, one point which has to be noted is that L-arg has been extensively described in the literature regarding its beneficial effects on glucose metabolism, endothelial function and cardiometabolic health and supplementing foods with L-arg could also offer cardiovascular protection [15]. 

A limitation of the present study is that amino acids content of the two designed biscuits could not be more differentiated regarding BCAAs and L-arg amounts since they were developed using flours and not pure substances. This fact may possibly make it difficult to clearly distinguish their results or highlight statistical differences. The significant results seem to be related more to L-arg content than BCAAs and the ideal case would be to have a biscuit enriched in proteins but with very low BCAAs vs L-arg content. In addition, the role of other biscuit components on the observed differences in metabolic responses and appetite ratings, such as the different amounts and composition of fat and fibers, cannot be underestimated.

## 5. Conclusions

In conclusion, the results of the present study showed that supplementation of wheat biscuits with plant flours originating from legumes and seeds, enriched either in L-arg or BCAAs, improved postprandial glucose responses compared to a common wheat biscuit. Additional analysis revealed stronger effects after consumption of the enriched biscuits regarding gastrointestinal hormone responses and subjective appetite sensations. Further investigation concerning enrichment with L-arg, an insulinotropic amino acid and GLP-1 secretagogue, becomes challenging especially if the cardioprotective effects of L-arg from the one side and the incriminating roles of BCAAs regarding their association with type 2 diabetes and insulin resistance on the other, are taken into consideration. The new products had very good organoleptic characteristics and were not less preferred than the common wheat biscuit.

In the present study, better and healthier alternatives in terms of snacking were made by simple materials with high nutritional quality and easy to find. Enrichment of foods with specific components of plant origin which positively affect glucose and gastrointestinal appetite-regulating hormone responses could provide a novel strategy for metabolic regulation and prevention of obesity.

## 6. Patents

Patent No: GR1010123 B1/ELBISCO S.A., HAROKOPIO UNIVERSITY OF ATHENS.

## Figures and Tables

**Table 1:** Characteristics of subjects who participated in the study.

Characteristic	Subjects	Range
n	30	-
Sex (male/female)	15/15	-
Age (years)	26.5 ± 5.3	18–39
Weight (kg)	77.8 ± 16.3	44.0–102.6
BMI (kg/m^2^)	25.7 ± 4.6	19.1–34.5
WHR	0.8 ± 0.2	0.68–1.03
Body fat (%)	25.5 ± 10.0	11.3–44.1
SBP (mmHg)	109.6 ± 22.8	100.3–141.0
DBP (mmHg)	67.8 ± 15.3	57.3–93.3
Fasting plasma glucose (mg/dL)	90.8 ± 7.2	72.6–100.0
Fasting serum insulin (μU/mL)	7.9 ± 5.8	2.1–22.0

n, number of subjects; BMI, body mass index; WHR, waist to hip ratio; SBP, systolic blood pressure; DBP, diastolic blood pressure. Values are presented as mean ± SD.

**Table 2:** Nutritional composition of the three tested biscuits.

Biscuit	Energy Content (kJ)	Available Carbohydrates (g)	Fat (g)	Protein (g)	Total Dietary Fibers (g)	Soluble Dietary Fibers (g)	Insoluble Dietary Fibers (g)	L-Arg (g)	L-Leu (g)	L-Ile (g)	L-Val (g)	BCAAs (g)	IAA (g)	TAA (g)	AAA (g)
*per* 100 g															
CB	1873.6	71.5	14.0	7.3	2.0	0.5	1.5	0.241	0.464	0.232	0.288	0.984	1.954	6.622	0.594
BCAAsB	1874.9	60.0	15.7	14.0	4.5	1.7	2.8	0.747	1.130	0.547	0.637	2.314	4.621	12.920	1.252
ArgB	1893.3	56.8	17.2	14.5	5.6	1.6	4.0	1.050	0.938	0.494	0.615	2.047	4.236	12.986	1.254
*per* 50 g of available carbohydrates															
CB	1310.5	50.0	9.8	5.1	1.4	0.4	1.0	0.169	0.325	0.162	0.201	0.688	1.366	4.631	0.415
BCAAsB	1564.0	50.0	13.1	11.7	3.8	1.4	2.4	0.623	0.942	0.456	0.531	1.928	3.851	10.767	1.044
ArgB	1665.5	50.0	15.1	12.8	4.9	1.4	3.5	0.924	0.826	0.435	0.541	1.802	3.729	11.431	1.104

CB, control wheat biscuit; ArgB, wheat biscuit enriched with L-arg; BCAAsB, wheat biscuit enriched with BCAAs. IAA, indispensable amino acids; TAA, total amino acids; AAA, aromatic amino acids. The portion size of the biscuits consumed was 69.9 g of CB, 83.4 g of BCAAsB and 88.0 g for ArgB.

**Table 3:** Incremental areas under the curve (iAUC) for glucose, insulin, gastrointestinal hormone responses and subjective appetite ratings (hunger, fullness and desire to eat) after ingestion of the three biscuits.

iAUC0-180 min	GS	CB	BCAAsB	ArgB
Glucose (mg*min*dL^−1^)	2508.3 ± 276.6	1307.7 ± 131.7 ^d^	739.5 ± 126.8 ^b,d^	861.7 ± 127.9 ^a,d^
Insulin (μIU*min*mL^−1^)	-	4333.5 ± 336.5	3329.8 ± 322.6 ^b,c^	4153.3 ± 341.3
Ghrelin (pg*min*mL^−1^)	-	−15,135.9 ± 2031.0	−18,409.8 ± 3320.5	−17,509.8 ± 2863.7
GLP-1 (pmol*min*L^−1^)	-	510.3 ± 112.5	1193.8 ± 245.7	1358.8 ± 205.3 ^a^
PYY (pg*min*mL^−1^)	-	2904.0 ± 655.6	5076.0 ± 1104.7	4141.5 ± 755.1
Glicentin (pg*min*mL^−1^)	-	4729.0 ± 1499.8	10,387.5 ± 2247.2	8328.8 ± 1943.3 ^a^
Hunger (cm*min)	−125.6 ± 28.3	−355.8 ± 44.0 ^d^	−499.8 ± 65.4 ^d^	−552.0 ± 60.4 ^a,d^
Fullness (cm*min)	149.9 ± 32.3	390.5 ± 60.6 ^d^	517.4 ± 65.9 ^b,d^	647.3 ± 65.1 ^a,d^
Desire to eat (cm*min)	−119.2 ± 26.3	−321.4 ± 53.8 ^d^	−506.3 ± 62.1 ^b,d^	−549.5 ± 48.8 ^a,d^

Values are expressed as mean ± SEM (*n* = 30). ^a^ *p* < 0.05 between ArgB and CB, ^b^ *p* < 0.05 between BCAAsB and CB, ^c^ *p* < 0.05 between BCAAsB and ArgB, ^d^ *p* < 0.05 compared to GS.

**Table 4:** Incremental areas under the curve (iAUCs) for L-arginine, L-isoleucine, L-leucine and L-valine after ingestion of the three biscuits.

iAUC0-180 min	CB	BCAAsB	ArgB
L-arginine (μmol*min*L^−1^)	829.7 ± 221.1	2036.5 ± 247.0 ^b^	2388.2 ± 234.3 ^a^
L- leucine (μmol*min*L^−1^)	634.9 ± 226.3	1435.3 ± 191.3 ^b^	995.1 ± 157.4
L- isoleucine (μmol*min*L^−1^)	352.6 ± 84.4	891.8 ± 107.4 ^b^	681.0 ± 100.7 ^a^
L-valine (μmol*min*L^−1^)	1088.2 ± 423.8	2164.6 ± 340.3	1608.8 ± 237.3

Values are expressed as mean ± SEM (*n* = 30). ^a^ *p* < 0.05 between ArgB and CB, ^b^ *p* < 0.05 between BCAAsB and CB.

**Table 5:** Organoleptic characteristics of the three biscuits.

Organoleptic Characteristic		BCAAsB	ArgB	CB
Appearance	Surface homogeneity	3.79 ± 2.20	3.93 ± 1.96	3.89 ± 2.47
	Appearance defects	4.07 ± 2.31	3.79 ± 2.06	3.21 ± 2.10
Color intensity		6.32 ± 1.70 ^a^	5.50 ± 1.45 ^a^	3.50 ± 1.67
Texture	Fracturability	5.00 ± 2.50	5.36 ± 2.58	5.04 ± 2.70
	Crunchiness	3.67 ± 2.25	3.89 ± 2.15	3.37 ± 2.02
	Hardness	3.22 ± 1.76 ^a^	2.93 ± 1.27 ^a^	5.15 ± 2.03
	Gumminess	5.22 ± 2.21 ^a^	4.33 ± 1.98 ^a^	3.67 ± 1.88
Odor		3.81 ± 2.42	3.59 ± 2.39	2.89 ± 1.89
Taste		3.19 ± 1.50 ^a^	4.08 ± 1.61 ^a^	5.89 ± 1.55
Aftertaste		4.04 ± 2.06	4.54 ± 2.16	4.35 ± 2.06
Overall acceptance		5.06 ± 2.00 ^a^	5.40 ± 1.77 ^a^	3.80 ± 2.39

Values are expressed as mean ± SD. ^a^ *p* < 0.05 compared to CB.

## Data Availability

Data available on request from the authors.
